# Toxin Diversity Revealed by a Transcriptomic Study of *Ornithoctonus huwena*

**DOI:** 10.1371/journal.pone.0100682

**Published:** 2014-06-20

**Authors:** Yiya Zhang, Yong Huang, Quanze He, Jinyan Liu, Ji Luo, Li Zhu, Shanshan Lu, Pengfei Huang, Xinyi Chen, Xiongzhi Zeng, Songping Liang

**Affiliations:** 1 The Key Laboratory of Protein Chemistry and Developmental Biology of Ministry of Education, College of Life Sciences, Hunan Normal University, Changsha, China; 2 State Key Laboratory of Pathogen and Biosecurity, Beijing Institute of Microbiology and Epidemiology, Beijing, China; 3 The State Key Laboratory of Genetic Engineering, Institute of Biomedical Science, Fudan University, Shanghai, China; Instituto Butantan, Brazil

## Abstract

Spider venom comprises a mixture of compounds with diverse biological activities, which are used to capture prey and defend against predators. The peptide components bind a broad range of cellular targets with high affinity and selectivity, and appear to have remarkable structural diversity. Although spider venoms have been intensively investigated over the past few decades, venomic strategies to date have generally focused on high-abundance peptides. In addition, the lack of complete spider genomes or representative cDNA libraries has presented significant limitations for researchers interested in molecular diversity and understanding the genetic mechanisms of toxin evolution. In the present study, second-generation sequencing technologies, combined with proteomic analysis, were applied to determine the diverse peptide toxins in venom of the Chinese bird spider *Ornithoctonus huwena.* In total, 626 toxin precursor sequences were retrieved from transcriptomic data. All toxin precursors clustered into 16 gene superfamilies, which included six novel superfamilies and six novel cysteine patterns. A surprisingly high number of hypermutations and fragment insertions/deletions were detected, which accounted for the majority of toxin gene sequences with low-level expression. These mutations contribute to the formation of diverse cysteine patterns and highly variable isoforms. Furthermore, intraspecific venom variability, in combination with variable transcripts and peptide processing, contributes to the hypervariability of toxins in venoms, and associated rapid and adaptive evolution of toxins for prey capture and defense.

## Introduction

Spider venoms contain mixtures of compounds with various biological activities that are used to capture prey or to defend against predators [1], [2]. Many of these molecules exert their effects by acting selectively and potently on ion channels (e.g., Ca^2+^, Na^+^ or K^+^ voltage-gated ion channels) in cells [3]–[9]. Owing to their extraordinary chemical and pharmacological complexity, spider venoms have elicited significant interest for use as tools to study neurophysiology and potential lead structures for pharmaceutics and insecticides [10]. To date, ∼40,000 spider species in 109 families, representing 400 million years of evolution, have been described, although venoms from only a few dozen species have been thoroughly investigated [11]. Spider venoms are highly complex mixtures containing, as a conservative estimate, over 300 toxin peptides per species. Hence, the total number of spider toxins could be over 11 million [11]. However, fewer than 1000 representative spider peptide toxins have been characterized and the mechanisms underlying toxin diversity are far from clear.

The majority of toxins found in spider venoms are small, bioactive and heavily post-translationally modified peptides. Disulfide-rich peptides (having two or more disulfide bonds) are known as CKTs (cystine knot toxins) and represent the majority of toxin peptides. Toxin peptides are synthesized in the venom gland as precursor proteins from a single gene comprising a highly conserved signal peptide, propeptide region and a highly variable toxin sequence. These peptides are classified into gene superfamilies according to sequence similarities of the signal peptide in the precursor. Despite the diversity of mature peptides, the molecular mechanisms of transcription preserve the cysteine residues, resulting in a high degree of conservation of the molecular scaffold. So far, over 10 different cysteine patterns have been identified in spider venom, with the number of residues ranging from four to fourteen [12]. Additionally, numerous post-translational modifications (PTMs), including hydroxylation of proline, valine and lysine, carboxylation of glutamate, C-terminal amidation, cyclization of N-terminal glutamine and glycosylation, contribute to the structural variety of the peptides [13], [14].

To date, 67 different toxin precursors from *Ornithoctonus huwena* have been identified, based on EST (Expressed sequence tag) sequencing of the cDNA library [15]. Separation of crude venom components using a combination of ion-exchange and reverse-phase high-performance liquid chromatography (HPLC) and 2D gel electrophoresis, followed by silver staining, revealed over 300 protein spots, 133 of which were detected with mass spectrometry [16], [17]. The large discrepancy between the gene and mass numbers detected in venom indicates that the low sensitivity of traditional transcriptomic approaches leads to the overlooking of rare sequences, which are transcribed at low levels. The recent availability of second-generation sequencing has facilitated the identification of several toxin-like peptides, significantly accelerating the pace of toxin discovery [18]–[20]. The 454 Life Sciences pyrosequencing technology is commonly used due to its high-throughput and accuracy comparable to traditional Sanger sequencing [21], [22]. We selected this approach, since it generates relatively long readable sequences (on average >300 bp) that encompass the full length of toxin precursors (60–120 amino acids). The technology allows direct identification of toxin precursors and avoids the errors inherent in the assembly of overlapping sequences (contigs) typically required for other second-generation technologies that generate shorter readable sequences (reads).

In the present study, 626 toxin precursors were unambiguously identified and classified into 16 different superfamilies, including six novel superfamilies and six novel cysteine patterns. A surprisingly large number of mutations, incomplete precursor sequences and aberrant sequences (i.e., interrupted or elongated cysteine patterns and highly variable isoforms, including deletions and elongations) were detected. The majority of these result from single amino acid changes and frameshifts. Interestingly, although most unusual toxin variants are expressed at very low levels, they may play an important role in the high rates of evolution of toxin genes within families. Moreover, along with alternative modes of peptide processing, these transcripts may explain the hypervariability of venom peptide and rapid evolution of bioactive peptides.

## Materials and Methods

### cDNA Library Construction and 454 Sequencing

The tarantula spider, *Ornithoctonus huwena,* is not a protected species and found widely in the Guangxi Province of China. Three tarantula spiders were collected for study. No specific permission was required for these locations/activities. Venom glands of *Ornithoctonus huwena* were obtained two days after being milked via electrical stimulation, and ground to fine powder in liquid nitrogen. Total RNA was extracted with TRIzol (Invitrogen, Carlsbad, CA, USA) and used to construct a cDNA library. Full-length enriched double-stranded cDNA was synthesized from pooled total RNA using the SuperScript First-Strand Synthesis System for RT-PCR (Invitrogen) and NEBNext mRNA Second Strand Synthesis Module (NEB), according to the manufacturer’s protocol, and subsequently purified using the QIAquick PCR Purification Kit (Qiagen USA, Valencia, CA). The DNA library was prepared from 300 ng samples using the manufacturer’s instructions (Rapid Library Preparation Method, Roche). Sequencing was performed on a Roche GS FLX Titanium sequencer.

### Sequence Assembly and Alignment

Sequence reads were trimmed by excluding low-quality regions using the NGen module of the DNAStar Lasergene software suite. Subsequently, assembly was performed with SeqMan pro (DNASTAR, USA) using high stringency *de novo* transcriptome assembly (100% identity between reads with 50 nucleotide sequence overlap). Similar 454 sequence reads were assembled into contigs using CLC Genomics Workbench 3 with its default parameters. Raw reads and contigs were uploaded in a proprietary web-based searchable database. As mentioned previously, such long sequence reads are likely to contain the full nucleic sequences of toxin precursors. Both raw reads and assembled contigs were identified as transcripts. Peptide sequences were identified from the transcript data using tBlastn. The E-value threshold of e ≤10^−5^ with a bit score >40 was recorded as a significant match for each query sequence. We analyzed the Blast results and used a home-made PERL script to classify representative sequences into five categories (‘Toxin-like’, ‘Putative toxin’, ‘Cellular Proteins’, ‘Unknown function’, ‘No Hit’). The identified peptide sequences were aligned using ClustalX 2.0.

### Gene Ontology Annotations

Functional characteristics of the transcriptome were predicted using BLAST2GO software [23] with the NCBI non-redundant protein database (cut-off e-value of ≤10^−5^) using EST contigs. Each contig with GI accession (NCBI) of the significant hits retrieved was assigned GO terms according to molecular function, biological process and cellular component ontologies at a level that provides the most abundant category numbers [24].

### Toxin Identification and Evolutionary Analyses

Known toxins often showed similar sequences, and the new toxin patterns expected a visitor. Moreover, the ‘Toxin-like’ sequences representing ‘no hits’ sequences with an abundance of cysteine residues, may encode new toxin peptides. Thus, ‘no significant hit’ sequences and those displaying high similarity with known toxins presented a huge treasury for toxin identification. Precursors encoded by these cDNA sequences were initially identified using NCBI BLAST, and those without significant BLAST results enriched with cysteine residues were identified as toxin precursors. Signal peptides were predicted with the SignalP 3.0 program (http://www.cbs.dtu.dk/services/SignalP/). The propeptide cleavage site was ascertained from the known start site of previously characterized mature toxins. Toxin-like proteins were grouped into different toxin families according to sequence similarity. All precursor sequences were aligned using ClustalX 2.0. The resulting alignment was imported into MEGA software to construct a phylogenetic tree with the neighbor-joining method [25], and bootstrap values estimated from 500 replicates.

The nucleotide sequences of superfamilies I, II and XVIII were aligned with ClustalX. The number of synonymous substitutions per synonymous site (Ds) and nonsynonymous substitutions per nonsynonymous site (Dn) were estimated using the original NeiGojobori model [26]. Fisher’s exact tests for positive evolution (based on the original Nei-Gojobori model) were performed using MEGA software.

## Results

### 454 Sequencing Statistics and Transcriptome Assembly

The mRNAs of six venom glands from tarantula *Ornithoctonus huwena* were extracted and sequenced using GS FLX technology (454/Roche) following the manufacturer’s protocol. Sequencing revealed a total of 123,922 reads (amounting to ∼42 Mb) with an average length of ∼327 bases per read, ranging from 40 to 836 bp. Raw sequencing data can be downloaded from SRA of NCBI using the accession number SRP039535. Overall, 80,908 reads were assembled into 4,224 contigs, while the rest remained as singletons. Both raw cDNA reads (>200 bp) and assembled contigs were collected for further analysis.

**Figure 1:**
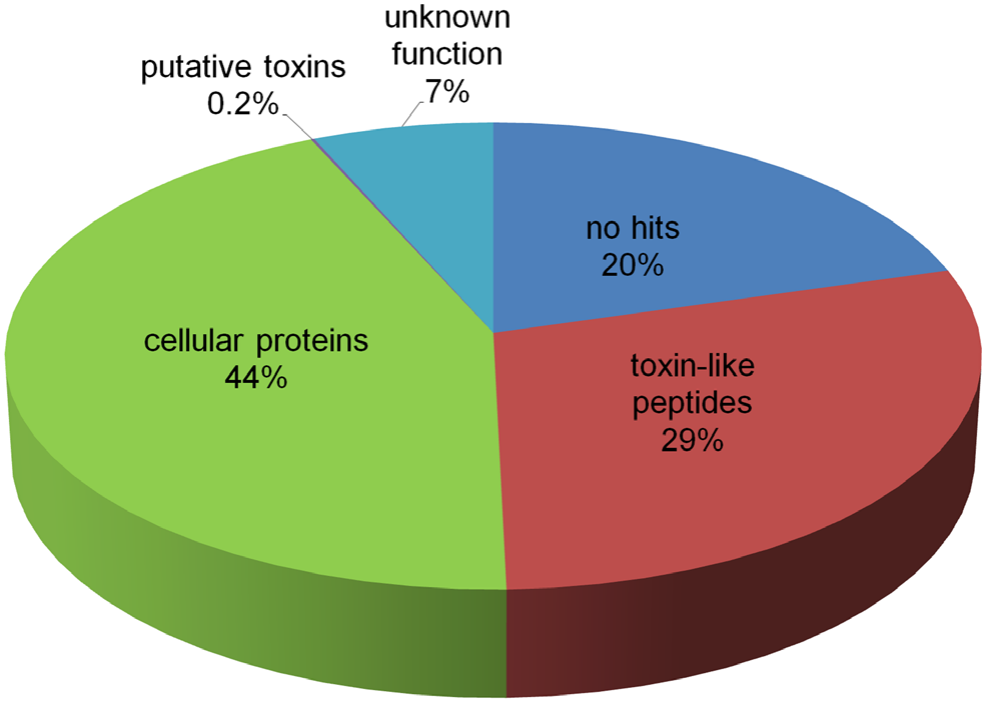
Classification of ESTs from *O. huwena* venom glands.

Representative sequences of each transcript were analyzed with the tBLASTn programs. Significant similarities were evident between 88,182 transcripts and proteins in the UniprotKB, Repbase and ToxRelDB databases. We observed high identity of 32,267 reads with toxin families, accounting for 29% raw reads. ‘Cellular Proteins’ includes transcripts coding for proteins involved in cellular processes (44%), including peptidases, cell signaling, cell structure and motility, metabolism and protein processing. For precursors with no significant BLAST results, cysteine patterns were extracted and empirically examined. The group ‘Putative toxins’ includes sequences rich in cysteine that display identity to toxins (0.1%). ‘Unknown function’ encompasses ESTs homologous to described sequences with no functional assessment or hypothetical genes (7%). About 20% reads were assigned to the “No Hit” category, indicating no match with currently known sequences. Results are summarized in Figure 1.

### Functional Annotation

**Figure 2:**
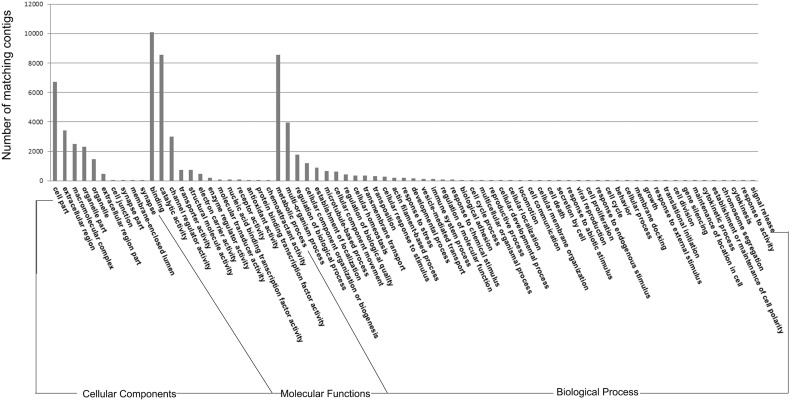
Gene ontology sorted annotations. Results are classified into Biological process, Molecular function and Cellular component at the second level, according to standard gene ontology terms. The different ontology categories are presented on the X-axis. Number of ESTs matching GO annotation terms (prior to clustering) are presented on the Y-axis. As more than 80% of the contigs failed to show association with GO terms, this analysis represents an annotation of the most conserved eukaryotic genes.

A search against public databases (nr/NCBI, Swiss-Prot+TREMBL/EMBL) revealed that ∼68.8% of all transcripts are associated with GO terms and further grouped into Molecular Functions (MF), Biological Process (BP) and Cellular Components (CC) at the second level according to standard gene ontology terms (http://www.geneontology.org). This finding was in agreement with previous data [13], [27], [28]. According to annotations from GO analyses (Figure 2), transcripts were categorized into 73 biological processes. “Metabolic process” indicating an important metabolic activity, was the most highly represented in *O. huwena* venom gland, similarly observed for snail *Conus consors* [20], [29]. For molecular function, binding and catalytic activities rank first, which is related to the high toxin peptide content in the venom gland of *O. huwena*.

Finally, four main categories of cellular components were identified, specifically, cell, extracellular region, organelle and macromolecular complex, all of which were mainly related to structural proteins involved in the secretion and transport of toxic compounds.

### Spider Toxin Transcript Analysis

Toxin peptides are the most abundant compounds of the spider venom gland. According to precursor sequence identity, 599 toxin precursors were produced from “spider toxin peptides” and 27 non-redundant precursor sequences from “putative toxin”, yielding 626 non-redundant precursor sequences in total. Interestingly, only a small fraction of the total peptide precursors found in assembled contigs were retrieved from raw data, indicating that genetic diversity is underestimated if only raw reads are analyzed. Several protein and enzyme sequences were additionally identified among the contigs.

**Figure 3:**
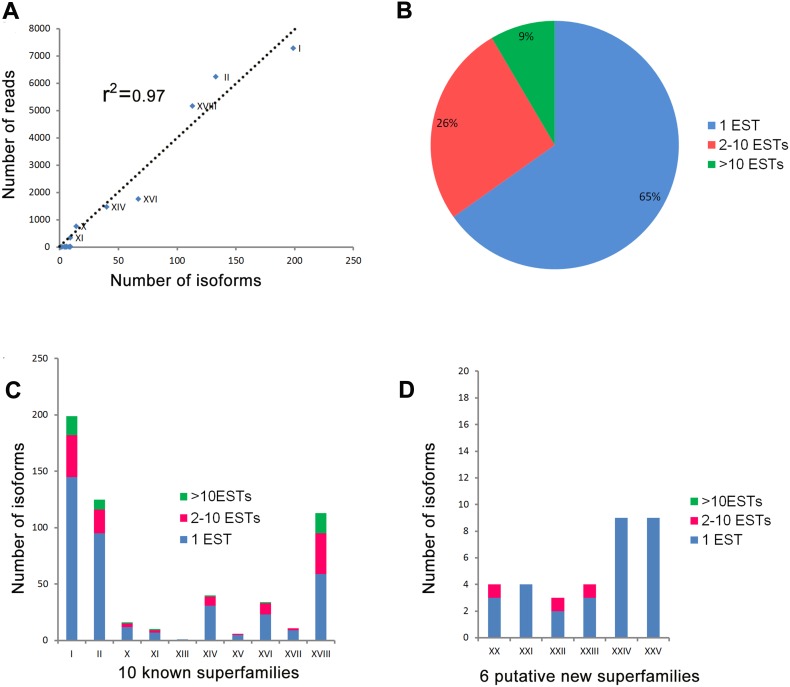
Isoforms and transcription levels of putative toxin peptides. A, The number of precursors and total number of reads per gene superfamily show an apparent correlation. The goodness of fit was R2 = 0.97, revealing a significant correlation between the two parameters. B, Variations in the transcriptional levels. Only 7% of the putative sequences had more than 10 cDNA reads, 27% had moderate cDNA reads of 2–10, and 66% were present as rare transcripts with only single cDNA read discovered for the full-length precursor. C, Isoforms of 10 known superfamilies. C, Isoforms of six putative new superfamilies.

Significant differences at the mRNA level were observed among the different toxin precursors. Precursors were most abundantly identified from superfamilies I, II and XVIII, corresponding to 9,286, 6,232 and 4,167 reads, respectively. This finding suggests that toxins more highly expressed at the mRNA level, and tend to be more abundant in the venom isoforms. Linear regression analysis (r^2^ = 0.97) indicated the highest number of reads in superfamilies with the largest number of precursors (Figure 3A). As shown in Figure 3B, 408 putative toxin precursors only had one cDNA read. These rare transcripts comprised ∼65% of the total retrieved putative toxin precursors. We additionally identified 53 high-level (>10 cDNA reads) and 165 low-level precursors (2–10 cDNA reads). The total number of precursors and cDNA reads for each superfamily are plotted in Figure 3C. Seven known superfamilies contained toxin precursors with high-level cDNA reads (>10 reads). Three of these (I, II and XVIII) had more than 9 high-level read precursors, while the remaining (X, XI, XIV and XVI) contained only one high-level read precursor. Precursors in superfamilies XV and XVII displayed low-level or very low-level expression. One precursor was identified in superfamily XIII with three cDNA reads. No high-level precursors were identified in the six putative new superfamilies, and only low or very low-level cDNA reads were observed (Figure 3D).

**Figure 4:**
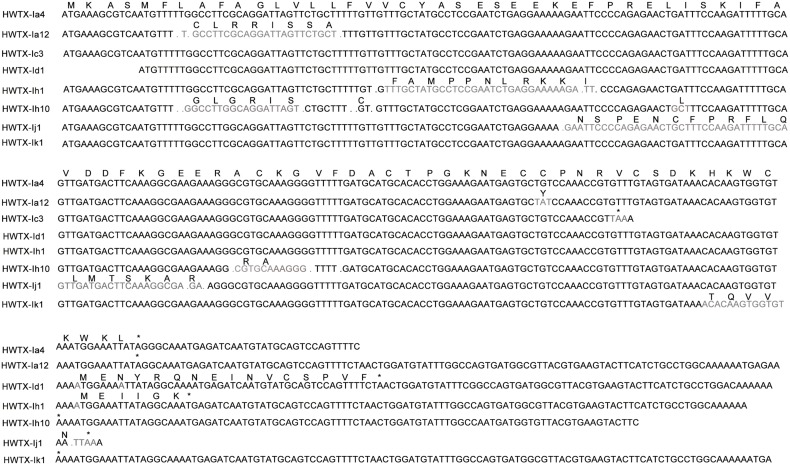
Comparison of the cDNA and precursor sequences of HWTX-Ia4 with the seven similar mutations.

Toxin precursors with very low or low-level expression often displayed high sequence identity with high-level precursors in the same family or superfamily. Analysis of gene sequences revealed that very low/low level precursors are variants produced by hypermutation, fragment insertion/deletion and mutation-induced premature termination and elongation. Eight toxin cDNA and precursor sequences are aligned in Figure 4. Mutation of the 100^th^ base “A” to “C” caused single substitution of a cysteine residue (HWTX-Ia12) and created an odd number of cysteine residues, leading to cysteine pattern disorder. Moreover, the absence of three bases resulted in frameshift mutation in the signal peptide region without altering the enzyme site and mature peptide region. Premature stop codons (HWTX-Ic3) produced truncated isoforms as well as truncated cysteine patterns. Frameshifts produced highly variable isoforms via deletions/additions in the C-terminal region (HWTX-Id1, Ih10 and Ij1and Ik1).

**Figure 5:**
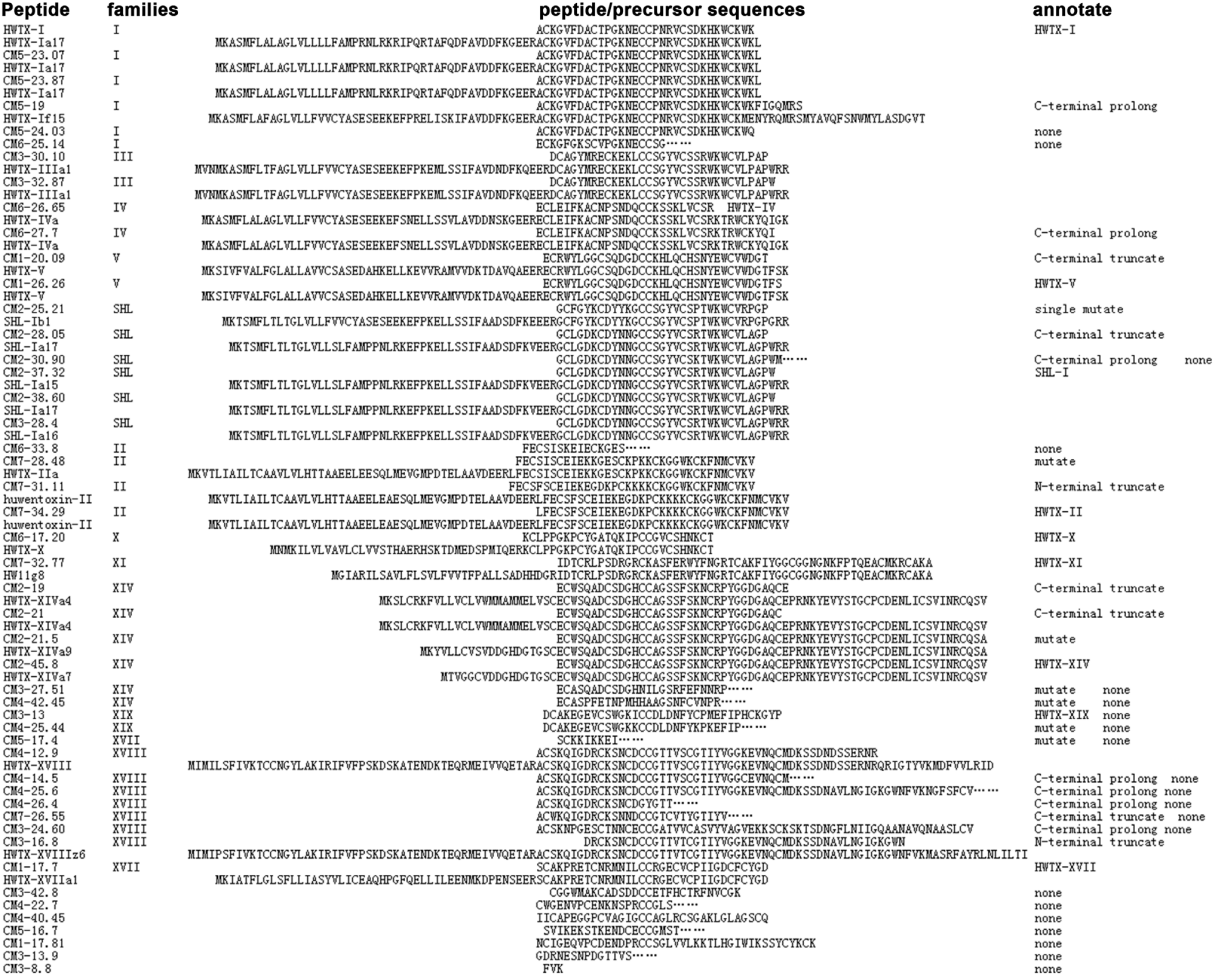
Full or partial sequences and biological activity of peptides from *O. huwena* found in both transcriptome and venom proteome.

Abundant toxin variations were additionally observed in venom. Among the 30 full and 17 partial sequences identified via Edman degradation [17], 40 (30 full and 10 partial) were clustered into eight superfamilies (12 families). As shown in Figure 5, single mutation (in CM5-24.03 vs. HWTX-I, CM7-28.48 vs. CM7-31.11 and CM2-21.5 vs. CM2-21.5) and C-terminal extension (in CM5-19 vs. HWTX-I, CM4-25.6 vs. CM4-12.9) were detected in venom that were possibly produced by diverse precursors. CM3-30.10 and CM3-32.87, generated from the same toxin precursor (HWTX-IIIa1), produced diverse isoforms via alternative cleavage and post-translational modifications (PTMs), previously reported as the major strategies underlying toxin diversity [13]. Different molecular masses were obtained for the same amino acid sequence via post-translational processing (in CM5-23.07 vs. CM5-23.87, CM6-26.65 vs. CM6-27.7 and CM2-37.32 vs. CM2-38.60).

Eight toxin superfamilies identified previously in venom from *Ornithoctonus huwena* were observed in the transcriptome, but their mutated isoforms showed considerable variations. Only 15 precursors and 24 putative mature peptides were detected with both approaches, probably caused by the well-known phenomena of intraspecific variations in many species [30]–[33].

### Toxin Precursors and Their Classification

**Figure 6:**
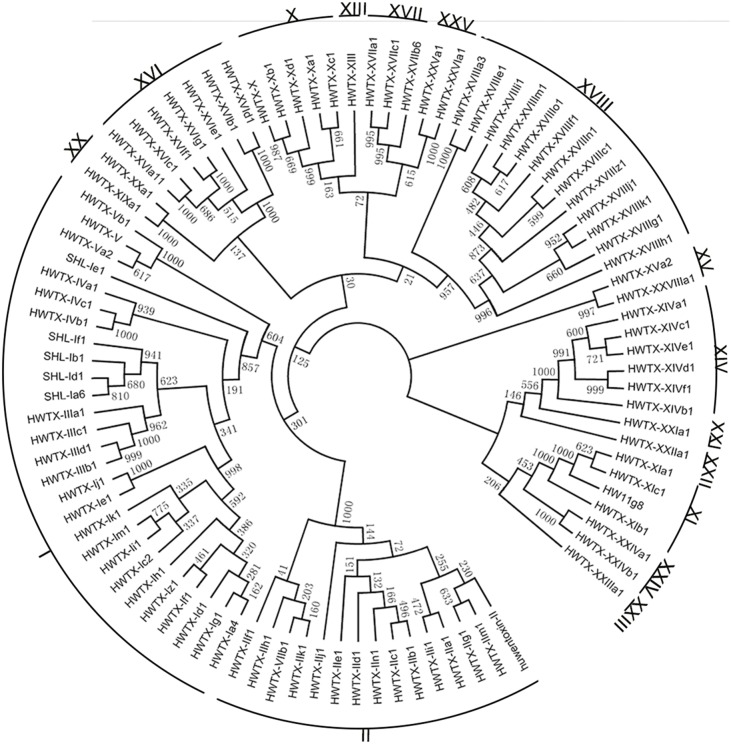
Phylogenetic tree of all venom peptide precursors in the HNTX superfamily from *O.huwena*.

Overall, 626 toxin precursors were categorized into 10 known and 6 putative new gene superfamilies (Table S1). The signal peptides and cysteine patterns are listed in Table 1. The phylogenetic tree shows the toxin precursors originate from three different clades. Members of the HWTX-I superfamily belong to one clade, the HWTX-XI, HWTX-XXII, HWTX-XXIV, HWTX-XIV and HWTX-XV superfamilies and HWT-XXIII, HWTX-XXI superfamilies belong to the second clade, and the others belong to the third clade. Each superfamily was further divided into several distinct families and subfamilies based on the identity of precursor sequences (Figure 6). In addition to the 15 known families, one novel family from the known superfamily XV and eight putative new families from six novel superfamilies (designated XX, XXI, XXII, XXIII, XXIV, XXV) were identified.

**Table 1:** Main features of toxins from the venom gland transcriptome of the tarantula *O.huwena*.

superfamily	signalpeptides	families	previousdata	cysteineframework	Functionidentity
I	MKASMFLAFAGLVLLFVVCYA	HWTX-I	HWTX-I	-c-c-cc-c-c- (I)	mammalian HVA Ca^2+^ channel initor and TTX-S Na^+^ channel inhibitor [34], [35]
		HWTX-III	HWTX-III	-c-c-cc-c-c- (I)	insect Na^+^ channel inhibitor [54]
		HWTX-IV	HWTX-IV	-c-c-cc-c-c- (I)	mammalian TTX-S Na^+^ channel inhibitor [51], [56]
		HWTX-V	HWTX-V	-c-c-cc-c-c- (I)	insect Ca^2+^ channel inhibitor [58]
		SHL	SHL-I	-c-c-cc-c-c- (I)	lectin [53]
II	MKVTLIAILTCAAVLVLHTTAA	HWTX-II	HWTX-II	-c-c-c-c-c-c- (II)	
		HWTX-VII	HWTX-VII	-c-c-c-c-c-c- (II)	
X	MNMKILVLVAVLCLVVSTHA	HWTX-X	HWTX-X	-c-c-cc-c-c- (I)	mammalian N-type Ca^2+^ channel inhibitor [36]
XI	MGIARILSAVLFLSVLFVVTFPALLSAD	HWTX-XI	HWTX-XI	-c-c-c-c-c-c- (II)	K^+^ channel inhibitor [37]
XIII	MKYAIVLCVIVIVVTVVRA	HWTX-XIII	HWTX-XIII	-c-c-cc-c-c- (I)	
XIV	MKVVLLVCLVWMMAMMELVSC	HWTX-XIV	HWTX-XIV	-c-c-cc-c-c-c-c-c-c- (VI)	
XV	MKHFASCIFSVLTVAICGVSQT	HWTX-XV	HWTX-XV	-c-c-cc-c-c-c-c- (III)	
		HWTX-XXVIII		-c-c-c-c-c-c-c-c- (V)	
XVI	MNTVRVTFLLVFVLAVSLGQA	HWTX-XVI	HWTX-XVI	-c-c-cc-c-c- (I)	
XVII	MKIATFLGLSFLLIASYVLICEA	HWTX-XVII	HWTX-XVII	-c-c-cc-c-c-c-c- (III)	
XVIII	MKLSLIIIATSLVIAVVA	HWTX-XVIII	HWTX-XVIII	-c-c-c-cc-c-c-c- (IV)	
XX	MKLSNFAVVLVGILFVSVPLFA	HWTX-XX		-c-c-cc-c-c-c-c-cc-c-c- (VII)	
		HWTX-XIX		-c-c-cc-c-c- (I)	
XXI	MLATFIVLFVPIFRNPLCCFQCQVYG	HWTX-XXI		-c-c-c-c-c-c-c- (X)	
XXII	MTWLLMVPLMLLSPLLQIAC	HWTX-XXII		-c-c-c-c- (IX)	
XXIII	MIVLGGMAFSVTSMVVACVV	HWTX-XXIII		-c-c-c-c-c- (VIII)	
XXIV	MVCATRRLIRLLSSSG	HWTX-XXIV		-c-c-c-c-c-c- (II)	
XXV		HWTX-XXV		-c-c-c-cc-c-c- (XI)	
		HWTX-XXVI		-c-c-c-c- (IX)	

### The HWTX-I Superfamily

**Figure 7:**
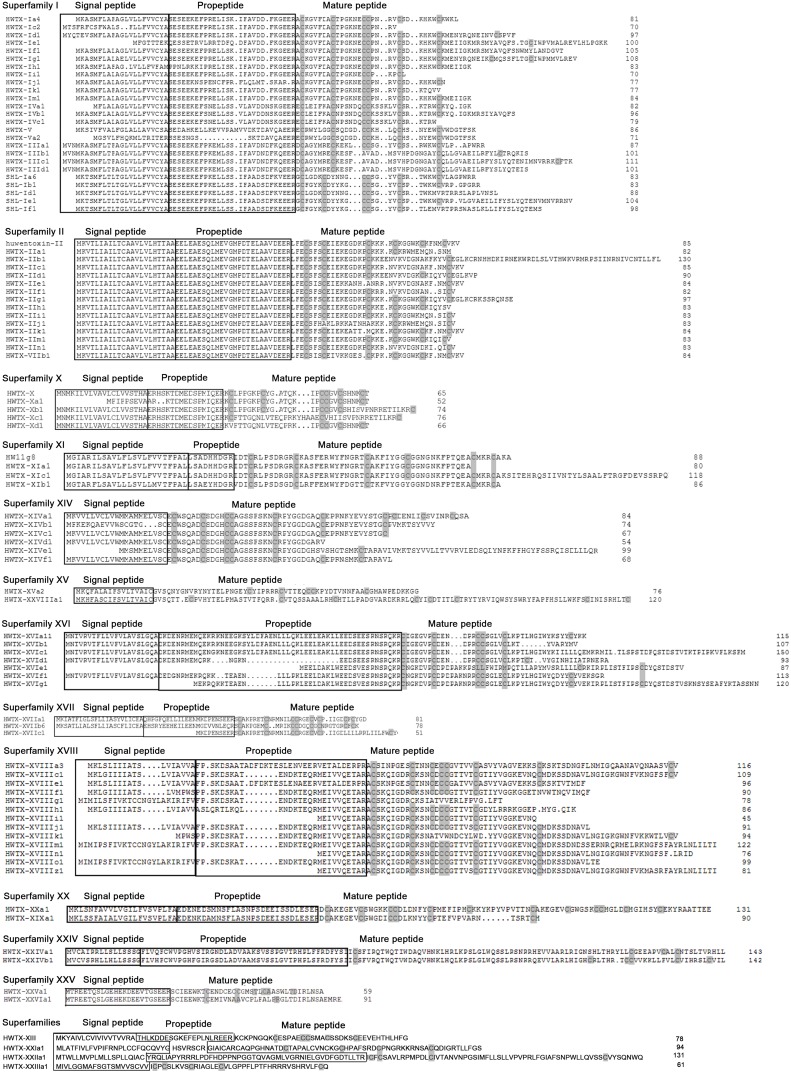
Sequence alignment of representative venom peptide precursors in superfamilies I-XXV from *O.huwena*. Signal peptides and propeptides are shown in framed boxes, and cysteines of mature peptides are highlighted.

In this superfamily, reads and toxin variants were the most abundant, with a total of 9,286 cDNA reads and 199 precursors, which clustered into five families (25 subfamilies: HWTX-I [a, c∼k, m], SHL-I [a∼f], HWTX-III [a∼d], HWTX-IV [a∼c] and HWTX-Va) (Figure 7). The majority of sequences in this superfamily showed high similarity. In particular, their precursors contained a highly conserved cleavage signal “CYASE” for signal peptides and consensus “GEER” cleavage signal for propeptide processing enzyme. Predicted mature peptides comprised 30 to 60 residues, three disulfide bonds and a classic Type I (C-C-CC-C-C) pattern. In total, 16 sequences displayed missing signal peptides, which was also a common phenomenon in the HWTX-XVI superfamily. Despite high sequence similarities among the five known families [15], the mature peptides of 20 novel subfamilies were highly variable. These novel subfamilies showed truncated or extended C-terminal regions produced by stop codon shifts and fragment insertion/deletion. Peptides of all known subfamilies were expressed at high levels, and those of most novel subfamilies at low levels (<50 reads), except HWTX-If and HWTX-Ih.

Family I contained 11 subfamilies (99 isoforms), including the known HWTX-Ia and HWTX-Ic and nine novel subfamilies. Among the known subfamilies, 31 and 8 isoforms were identified, respectively, although precursors HWTX-Ia4 and HWTX-Ia9 have been detected previously in *O. huwena*. HWTX-Id, HWTX-Ie and HWTX-Ig contained a long cysteine pattern with one or two additional residues in the C-terminal region, compared with HWTX-Ia. The same cysteine arrangement, -C-C-CC-C-C- (cysteine pattern I), was observed in HWTX-If, HWTX-Ih and HWTX-Im. We speculate that these variants potently inhibit high voltage-activated (HVA) Ca^2+^ channels [34], [35]. HWTX-Ih showed a short pattern with cysteine residues missing at the C-terminal, which would break III-VI disulfide bonds. This phenomenon was additionally observed in the known toxins HWTX-Ib and HWTX-Ic [15].

Overall, 16 precursors belonging to the HWTX-III family were classified into one known and three novel subfamilies. HWTX-IIIa was the known subfamily with three toxin precursors, but only one (HWTX-IIIa1) has been detected previously. HWTX-III [b∼d] contained an extended C-terminal region and an impaired disulfide bridge with the double cysteine motif missing.

We identified 14 precursors in the HWTX-IV family, which were clustered into three subfamilies. Two novel subfamilies (HWTX-IVb and HWTX-IVc) showed C-terminal elongation and truncation, respectively. The missing cysteine in HWTX-IVc may lead to loss of inhibitory activity of the peptides on the neuronal tetrodotoxin-sensitive voltage-gated sodium channel. Mature sequences from short and long precursors displayed strikingly similar primary sequences. Accordingly, we speculate that C-terminal diversification results from a simple alteration of the stop codon.

Only one subfamily (HWTX-Va) was detected in HWTX-V containing 10 precursors. These are variants of HWTX-V that show high sequence similarity with HWTX-V, and specifically inhibit high voltage-activated calcium channels in adult cockroach dorsa.

SHL-I was additionally identified as an abundant toxic component with 60 toxin precursors. The C-terminal mutation in SHL-Id and SHL-If destroyed the disulfide bridge via alteration of the sixth cysteine residue to valine.

### The HWTX-II Superfamily

HWTX-II was the second most abundant superfamily with 133 isoforms, accounting for 24% toxin reads, and classified into three families (HWTX-II, HWTX-VII and HWTX-XI). In addition to the three known subfamilies, eleven new subfamilies displayed high identity with HWTX-II. HWTX-II [b, g, m], HWTX-VIIb and HWTX-XIc exhibited the same cysteine pattern II (“-C-C-C-C-C-C-”) as HWTX-II, with I–III, II–V and IV–VI disulfide connectivity and a DDH three-dimensional structure motif. Despite the C-terminal elongation, these could be the neurotoxins as ion channels antagonists. However, HWTX-II [c∼f, h∼k, n] had one to three cysteine residues missing, which may significantly affect disulfide bond formation. HWTX-VII displayed high sequence identity (about 81%) with the HWTX-II family, implying similar molecular folding and bioactivity (Figure 7).

### The HWTX-X Superfamily

This superfamily contained 14 members and was classified into four subfamilies. The three new subfamilies showed high sequence identity with the known peptide (HWTX-X) (Figure 7). HWTX-Xb shared the same cysteine pattern I (“-C-C-CC-C-C-”) as HWTX-X, and an expanded C-terminal region of about 20 residues that did not affect its biological function as an N-type Ca^2+^ channel inhibitor [36]. The main motif “IPCCGVCSHNKCT” in HWTX-Xc was modified to “YHAAECVHIISVPNRRETILKRC” with a double cysteine residue missing. The ICK motif (inhibitor cystine knot motif) in HWTX-Xc was disrupted and the rest of the cysteine residues could form two disulfide bonds in a way of independent assortment. This phenomenon was additionally observed in the HWTX-Xd subfamily. We speculate that the mutation contributes significantly effect to the functional diversity of HWTX-X.

### The HWTX-XI Superfamily

HWTX-XI is a serine protease inhibitor consisting of 55 residues with three disulfide bridges [37]. Previous results indicate that HWTX-XI follows the classical Kunitz architecture formed by three disulfide bridges with a linkage pattern of I-VI, II-IV, and III-V [15]. Overall, nine precursors were detected for the HWTX-XI superfamily, although only Huwentoxin-11g8, a known isoform, was expressed at a level higher than 50 reads [37].

HWTX-XIb contained two peptides lacking the II-IV disulfide bond, with tyrosine replacing the fourth cysteine. Members of this group have been designated ‘sub-Kunitz type’ toxins (cysteine pattern VIII). The sub-Kunitz type toxin has a Kunitz motif formed by the remaining two disulfide bridges [38]. In the bovine pancreatic trypsin inhibitor (BPTI) reduction experiment, native-like conformation and trypsin inhibitor activity remained for BPTI without the disulfide bond [39]. Similarly, HWTX-XIa lacked two cysteine residues in the C-terminal region. The final member of the HWTX-XI superfamily was HWTX-XIc. Precursor peptides in this subfamily contained a very long mature peptide region (>110 residues) with the ICK motif (Figure 7).

### The HWTX-XIV Superfamily

The HWTX-XIV superfamily contained 40 members, which were further classified into six subfamilies. The signal peptides displayed high similarity, although mature peptides contained a highly variable C-terminal region, and all were processed from precursors containing no propeptide sequences (Figure 7). The sequence identities of the mature peptides of HWTX-XIV [b, c] were 76.2% and 79.4%, compared to HWTX-XIVa1, respectively. However, the C-terminal regions lacked two cysteine residues, caused by the removal of two “T” bases and insertion of one “A” base in the cDNA sequence, respectively. Both cDNA sequences of HWTX-XIV [d, f] lacked three cysteines, resulting from the insertion of two ”G” and absence of two “A” bases. The mature peptide of HWTX-XIVe showed 43.1% similarity with HWTX-XIVa and cDNA sequence identity of 85.1%, indicating different bioactivities. Although this mutation may not be structurally significant, it contributes significantly to the considerable diversity of toxins peptides in venom gland.

### The HWTX-XV Superfamily

The HWTX-XV superfamily included two families with six members. Four precursors were identified for the known HWTX-XV family. HWTX-XVa5 displayed a truncated pattern with two cysteine residues missing at the C-terminal region.

HWTX-XXVIIIa was identified as a new subfamily devoid of the propeptide region, with a similar signal peptide as HWTX-XVa (Figure 7). Members in this family displayed the same cysteine pattern V (“-C-C-C-C-C-C-C-C-”), distinct from other known toxins in this spider. Moreover, the mature HWTX-XXVIII peptide contained more than 100 residues, and was considerably longer than HWTX-XV.

### The HWTX-XVI Superfamily

In the HWTX-XVI superfamily, 66 precursors were derived from seven subfamilies. We identified 27 precursors for the known HWTX-XVIa subfamily, none of which had been detected previously. From the precursors of six novel subfamilies, mature peptides are diversified through a C-terminal drift. HWTX-XVIb contained a cropped C-terminal region and HWTX-XVId displayed C-terminal elongation, compared with HWTX-XVIa. HWTX-XVI [c, e∼g] with a longer mature peptide, showed low cDNA sequence identity with the HWTX-XVIa family. The HWTX-XVI [c, d] and HWTX-XIVe subfamilies only contained five and four cysteine residues, respectively, which could disrupt the cysteine pattern (Figure 7).

### The HWTX-XVII Superfamily

Seven precursors were identified in the HWTX-XVII superfamily, with three subfamilies distinguished based on sequence similarity of the mature peptide. Two known subfamilies, HWTX-XVIIa and HWTX-XVIIa, have been detected previously in the cDNA library of *O. huwena* [15] but only two precursor sequences identified in both our transcriptome study and previous work. Precursors of the novel subfamily, HWTX-XVIIc, contained a much shorter signal peptide “MKDPENSEER” and had no propeptide sequence (Figure 7). Their mature peptides shared low sequence homology with the HWTX-XVIIa family, with a significantly shorter cysteine pattern (“-C-C-CC-C-C-”).

### The HWTX-XVIII Superfamily

HWTX-XVIII was the third most abundant superfamily, including 113 sequences. Two known subfamilies, HWTX-XVIIIa and HWTX-XVIIIc, showed higher expression than 100 reads, and ten novel subfamilies displayed lower expression (except HWTX-XVIIIj, HWTX-XVIIIk and HWTX-XVIIIz) (Figure 7). HWTX-XVIII [e∼j and o] were the truncated toxins of HWTX-XVIIIa. HWTX-XVIII [k and z] showed different C-termini with cysteine mutations. Loss of cysteine residues led to an odd pattern in these subfamilies, caused by deletion or insertion in the cDNA sequences. This phenomenon was also observed in HWTX-XVIIIc2 and HWTX-XVIIIc3 [15].

### The HWTX-XX Superfamily

Six precursors were detected in the novel superfamily HWTX-XX, with two families (HWTX-XIX and HWTX-XX) distinguished based on sequence similarity of the propeptide region (Figure 7). The mature peptide of HWTX-XIX contained 40 amino acids with a conservative cysteine pattern “DCX_6_CX_5_CCX_6_CX_14_CX” (X is any amino acid), which displayed significant homology with the ICK motif. Other than the highly conserved arrangement of cysteine residues, HWTX-XIX had no obvious sequence similarity with other toxin peptides. The mature peptide of HWTX-XX was similar to DkTx with the cysteine pattern VII (“-C-C-CC-C-C-C-C-CC-C-C-”) with the largest number of cysteines in HWTXs. The presence of two double cysteine motifs in this pattern implies that HWTX-XX contains the two disulfides through knots that are separated by a short linker. HWTX-XXa3 was a truncated toxin of HWTX-XXa1 with the codon terminated earlier.

### The HWTX-XXIV Superfamily

Nine precursors were identified in this superfamily, which displayed very low expression (one read). The precursor peptide of the HWTX-XXIV superfamily included identical signal peptide and propeptide, but the mature regions in HWTX-XIVa were highly variable and different from those of known toxins. A consistent cysteine pattern “XCX_50_CX_9_CX_7_CX_2_CX” (X is any amino acid, n is any number) (Figure 7) was observed, with the same number of cysteine residues as the sub-Kunitz motif [40].

### The HWTX-XXV Superfamily

This novel superfamily contained 10 precursors that clustered into two subfamilies (XXV and XXVI). Members of HWTX-XXV did not contain a signal peptide and the propeptide sequence was identified as “MTREETQSLGEHEKDEEVTGSEER”. The mature peptide of the HWTX-XXV family contained 75 residues with a consensus cysteine pattern XI (“-C-C-C-CC-C-C-”) containing an odd number of cysteine residues instead of the usual even number expected for the formation of internal disulfide bonds. The HWTX-XXVI sequence exhibited the same signal peptide and propeptide as HWTX-XXV, but the cysteine pattern IX (“-C-C-C-C-”) was considerably shorter.

### HWTX-XIII, HWTX-XXI, HWTX-XXII and HWTX-XXIII Superfamilies

Four novel superfamilies (XIII, XXI, XXII and XXIII) displayed low-level expression and contained one, five, two and four precursors respectively. HWTX-XIII was composed of 77 residues, and the hypothetical mature peptide contained 37 residues with three disulfide-bridged motifs [15]. Signal peptides of HWTX-XXI superfamily members were similar, with identical cysteine patterns (“X_4_CX_2_CX_9_CX_5_CX_2_CX_2_CX_7_CX_n_”, whereby X is any amino acid, n is any number) but highly variable mature peptides. The mature peptide of HWTX-XXIa4 contained 131 residues. A single point mutation may destroy the disulfide bridge by changing the fourth cysteine residue to methionine. Members of the HWTX-XXIII superfamily showed high sequence identity, except for the presence of different residues (leucine or glutamine) at the end of the mature peptide (Figure 7). Precursors of the HWTX-XXII superfamily had identical signal peptides and propeptides with a cysteine pattern of “-C-C-C-C-” (IX), which was not common in other spiders. This positional pattern of cysteines is also found in Kappa-hefutoxin-1, a potassium channel inhibitor that belongs to the short scorpion toxin superfamily (kappa-KTx family).

### Different Evolution Strategies of Superfamilies I, II and XVIII

**Figure 8:**
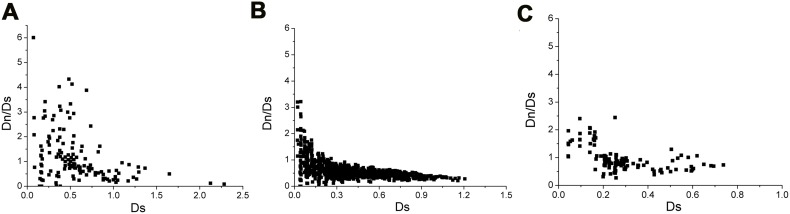
Nonsynonymous versus synonymous substitutions in superfamilies I, II and XVIII. The number of nonsynonymous substitutions per nonsynonymous site (Dn) over the number of synonymous substitutions per synonymous site (Ds) for interfamilial comparisons of (A) superfamily I, (B) superfamily II, (C) superfamily XVIII precursor peptide-encoding domains. Dn/Ds>1 (positive or diversifying selection), 1>Dn/Ds>0.27 (lack of constraints) and Dn/Ds<0.27 (purifying selection).

Propeptides displayed high sequence similarity in each superfamily. The contrasting high diversity of mature peptides may be a strategy of toxin evolution. To further explore evolutionary patterns, the Dn/Ds ratios from precursor peptides were examined. The majority of Dn/Ds ratios of superfamily II and XVIII were located in the region lacking constraints, indicating neutral evolution (Figure 8. B, C). Few of the Dn/Ds ratios within small genetic distances (<0.2) were significant (>1) implying positive selection evolution. The decrease in ratio at larger genetic distances was attributed to saturation. Half the Dn/Ds ratios of superfamily I were significant (>1), showing more positive selection evolution than superfamilies II and XVIII (Figure 8A). Additionally, we examined the null hypothesis of neutral evolution with Fisher’s exact test (cut-off of 0.05) with the Nei and Gojobori model [26]. No significant results were observed with superfamilies I, II and XVIII to reject a null hypothesis. In the HWTX-II superfamily, positive selection was detected in a clade containing four toxins [a, i, g and m], but not other clades. In the HWTX-XVIII superfamily, positive selection was detected in a clade containing three toxins, HWTX-XVIII [h, j and k], while HWTX-XVIIIc was identified in another clade.

## Discussion

As reported previously, most spider peptide toxins are identified at three different levels: transcriptomic, peptidomic, and genomic [13], [27], [41]–[44]. However, the information obtained from venom gland cDNA libraries and protein sequencing is limited and may be biased towards the components expressed in high abundance [11], [45], [46]. Recently, next-generation sequencing technology resulted in an explosion of sequence data for toxin transcripts, both in terms of number and breadth [20], [47]–[49]. The new 454 life Sciences pyrosequencing technology generates relatively long readable stretches (on average >300 bp) that cover the full length of toxin precursors (60–120 residues). This approach allows the direct identification of peptide precursors and avoids errors inherent to the assembly of overlapping sequences (contigs) typically required for other second-generation technologies that generate shorter reads.

In this study, 123,922 Expressed Sequence Tags (ESTs) were obtained using 454 high-throughput sequencing technology. A total of 626 putative toxin precursors (containing 398 mature peptides) were unambiguously retrieved from transcriptomic data, among which 85 toxin subfamilies within ten known superfamilies and six new superfamilies were analyzed in detail. In the representative precursors of all superfamilies from *O.huwena*, primary structure analysis disclosed the presence of the PQM motif (Processing Quadruplet Motif) [45], except for families XI, XIV, XXIII, XXIV lacking the propeptide.

All mature peptides in superfamilies contained 4–12 cysteine residues, which could be classified as one of 11 types (pattern I- XI). These include five cysteine patterns observed earlier in *O.huwena* and six new patterns. Cysteine pattern I was the most common. This pattern folds into the highly stable ICK motif found in a wide range of bioactive peptides in both animal and plant kingdoms [36], [50]–[54], indicating early evolution in the speciation of spider. As shown in Table 1, nine families shared cysteine pattern I and displayed voltage-gated sodium, potassium and calcium channel inhibition and lectin activity, indicating that ICK motif toxins are central to the success of spider evolution [36], [55]–[58], including *O.huwena*, which uses the multiple target strategy to disrupt neuronal functions of prey and/or predators. Six novel cysteine patterns have been identified for the first time in *O.huwena*, including three odd patterns. Cysteine pattern VII (“-C-C-CC-C-C-C-C-CC-C-C-”) with 12 residues, found in mature peptides of family XX, was the longest cysteine arrangement in *O.huwena*. Two contiguous cysteine residues were present twice in these sequences (-C3C4- and -C9C10-). These sequences showed high similarity with DkTx, which forms two independently folded domains connected by a kinked tether [59]. Cysteine pattern V (“-C-C-C-C-C-C-C-C-”) was conserved in scorpion toxin BmKAEP2 [60], kurtoxin [61], L-4CC-Alpha toxin [62], and MkTx I [61], [63], [64]. Scorpion toxin displays a cysteine arrangement with I–VIII, II–V, III–VI, IV–VII disulfide bonding patterns. Moreover, other cysteine patterns of eight residues were evident. Delta-MSTX-Mb1a from Eastern mouse spider contains the cysteine pattern “-C(X_4–8_)C(X_4–8_)CCC(X_2–3_)C(X_9–15_)C(X_9–15_)C-” and a I–IV, II–VI, III–VII, V–VIII disulfide bonding pattern [65]. The cysteine arrangement “-C(X_4–8_)C(X_4–8_)CC(X_1_)CC(X_4–8_)C(X_4–8_)C-” with a I–IV, II–VI, III–VII, V–VIII disulfide bonding pattern has been reported in Iota-conotoxin RXIA (r11a) and I-superfamily conotoxins from *Conus radiatus* [66], [67]. Moreover, short disintegrin CV from Sahara sand viper and Disintegrin pyramidin-A from *Echis pyramidum* leakeyi share the cysteine pattern “-C(X_4–8_)CC(X_2–3_)C(X_4–8_)C(X_9–15_)C(X_4–8_)C(X_1_)C-” with a I–IV, II–VI, III–VII, V–VIII disulfide bonding pattern [68]. IX, X and VIII contained an odd number of cysteine residues instead of the usual even number expected for the formation of internal disulfide bonds. The potassium channel toxin-like peptide, MeuKTX-1 [69], displays a cysteine pattern similar to that of X. The cysteine pattern VIII was a classical sub-Kunitz type motif, similar to that observed previously in *H. hainanum*. The cysteine pattern IX (“-C-C-C-C-”) was also present in the mature peptides of families XXII and XXVI. This structure is rarely found in spider venom, but frequently observed in conopeptides [19], [20]. The various cysteine patterns may underlie the diverse functions of toxin peptides. Some spiders use neurotoxins as the main weapons to specifically target the nervous system for killing or paralyzing prey. Other assistant toxins, such as channel TRPV inhibitors or lectin, may enhance the toxicity of venom by binding to their targets.

In this study, we estimated the level of precursor transcription from the number of reads for each transcript. Remarkably, the transcriptome of the *O.huwena* venom gland was predominated by three toxin superfamilies (I, II and XVIII), both in terms of the mRNA level and number of peptide isoforms present, suggesting an important role in prey capture and/or defense. Indeed, these three superfamilies account for at least 81% of all readable toxin cDNA sequences. This level of transcription for superfamilies was also accompanied by a high number of isoforms (71.2%). Homology analysis revealed important clues regarding toxin evolution (Figure 8). Superfamilies I, II and XVIII have been found in several genera, indicating that these toxins may have been derived from different ancestors. The Dn/Ds ratio exploring evolutionary patterns further showed that superfamily I has more positive selection than II and XVIII. In contrast, most toxins in the novel superfamily were found in very low abundance, indicative of assistant roles in venom. Moreover, the transcript levels may reflect those of the corresponding toxin peptides found in crude venom to a certain extent. For instance, HWTX-Ia transcript was the most highly expressed in the *O.huwena* venom gland, and its corresponding peptide, HWTX-I, the most abundant component detected in venom [50]. While the majority of toxin peptides are produced by precursors with high-level reads, surprisingly, some peptides are produced by precursors with low- and extremely low-level reads, and even some that could not be confirmed at the transcriptome level. For example, superfamily-I isoforms, CM5-24.03(HWTX-Ia) and CM3-13, previously discovered in the venom of *O.huwena* were not detected in the venom gland transcriptome. The results suggest that these toxin peptides are expressed randomly in venom, depending on environmental changes. In contrast, sequences belonging to superfamilies X and XI, which target N-type Ca^2+^ and K^+^ channels, were observed at low transcription levels, but reasonably abundant at the peptide level. One theory is that evolutionary pressures influence the level of expressed toxin peptides [70], but we believe that this is only a partial explanation, since evolution is a lengthy process that cannot create such differences between individuals within a short time. As described previously, highly expressed toxins detected with the cDNA library, as well as all full-length sequences and majority (10/17) of partial sequences identified via Edman degradation were detected via 454 sequencing, implying that highly expressed transcripts and abundant peptides are conservative constituents of venom. Unexpectedly, abundant transcriptomic profile differences were detected with the 454 transcriptome and cDNA library approaches. The dynamics of transcriptional changes demonstrate that venom samples of individual *O.huwena* are not constant in composition and vary dramatically with time. Moreover, this considerable variation may be associated with changes in diet, environment or replete/milk [30], [32], [33], [71]. Evolutionary innovations are proposed to be a result of infidelity of transcription and heterogeneity inherent to most biological processes, leading to genetic and phenotypic variations [72].

The large number of toxin variants are identified at transcriptional level is an expected finding. Notably, the majority of these modified mRNAs are also transcribed at low levels, which explains why these rare sequences have eluded detection in previous studies using traditional transcriptomic approaches. Toxin gene sequences include single base mutations, deletions, insertions and frame shifts, generating amino acid mutations, insertions and deletions, alternative cleavage sites and cysteine patterns, and highly variable isoforms within families that could be identified at the peptide level. Together with the variable peptide processing described previously, this mechanism contributes to the hypervariability of venom peptides and their ability to evolve rapidly. Post-translational modifications are relatively common in spider venom peptides [13], [73], [74], and play an important role in the modulation of biological activity. The commonly observed C-terminal amidation and trimming may generate additional isoforms. Based on the present results, we propose that both genetic and post-translational modifications contribute to overall toxin diversity. Therefore, background genetic diversity, in addition to the generation of highly variable transcripts and peptide processing, appears to underlie overall venom peptide diversity. In conclusion, the numerous toxins in spider venom and modification mechanisms have enabled the spider to adapt to its specific environment during evolution.

## Supporting Information

Table S1 **626 toxin precursors were categorized into 16 gene superfamilies.**
**(XLS)**
